# Virtual intravascular visualization of the aorta for surgical planning in acute type A aortic dissection

**DOI:** 10.1016/j.xjtc.2024.03.017

**Published:** 2024-03-28

**Authors:** Maycee Gielow, Rabin Gerrah

**Affiliations:** a Department of Surgery, Good Samaritan Regional Medical Center, Corvallis, Ore; b Department of Cardiothoracic Surgery, Stanford University Cardiovascular Institute, Stanford University, Stanford, Calif

**Figure:**
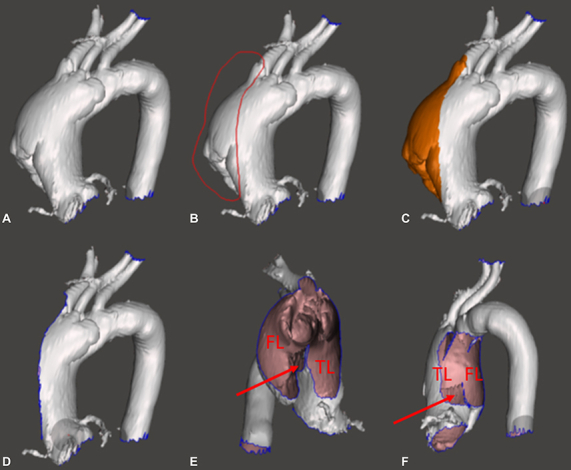
Virtual endoscopy of aortic dissection: Delineation of the flap, true lumen, and false lumen.

**Central Message**

Virtual intravascular endoscopy by the surgeon before the surgery for type A dissection provides custom planes for accurate assessment of the dissection flap to assist with surgical planning.

Acute type A aortic dissections (TAAD) often require emergency surgery. The goal of surgery for this life-threatening condition is to exclude the entry point of dissection and to reclaim integrity of blood flow in the true lumen of the aorta. The extent and type of surgery depends on the proximal and distal extension of the dissection, and involvement and function of the aortic valve and coronary arteries. Computerized tomography (CT) is the modality of choice to confirm the diagnosis and to aid in surgical planning; however, in some cases, exact localization and the morphology of the intimal tear and involvement of the aortic valve leaflets and arch branches are not clearly visualized in standard images (Figure 1). Additional imaging techniques such as 3-dimensional (3D) reconstruction can further delineate the exact anatomy of the dissected aorta; however, these techniques are not readily available in emergency settings.

**Figure 1:**
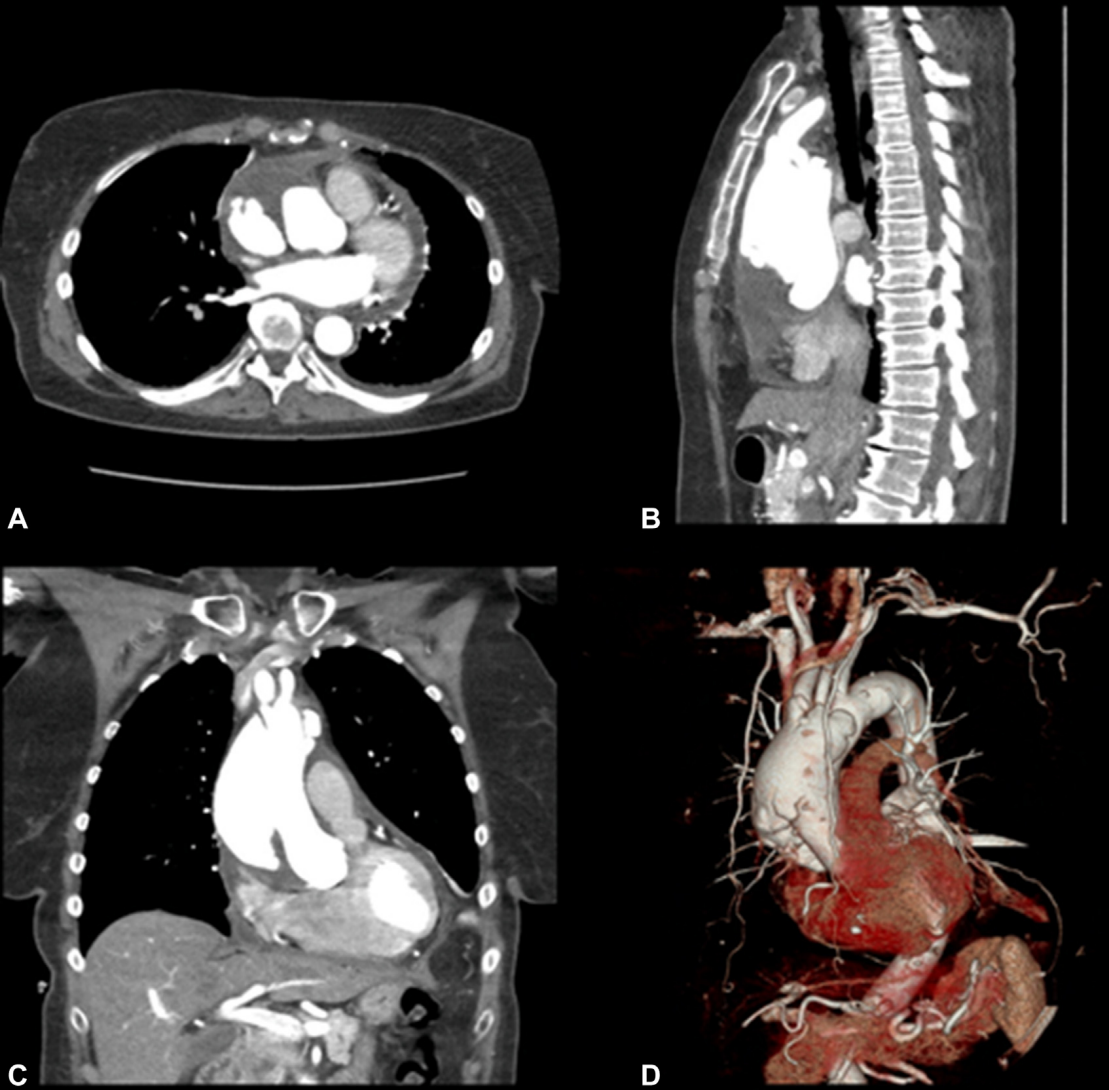
A 3-dimensional (*3D*) reconstruction image as part of preoperative imaging provided by the radiology department. Sample images from preoperative computerized tomography study providing routine 3 standard planes and the 3D image reconstructed from these images. In everyday practice, the diagnosis and surgical planning are based on these images. A, Axial view. B, Sagittal view. C, Coronal view. D, 3D reconstruction.

Virtual intravascular endoscopy (VIE) is a useful tool for realistic endoluminal visualization. In TAAD it allows an inside view of the location and extension of the tear, as well as provides a 3D view of the flap. Routine application of this technology in TAAD has been limited because it relies on technical expertise, it is time-consuming, and it requires additional resources not always available in an emergency setting.

In this article, we present a fast and simple way of VIE in a do-it-yourself manner by surgeons for surgical planning of acute aortic dissection. This process utilizes 2 open-source programs and takes approximately 10 to 15 minutes. The advantage of VIE by a surgeon versus a radiologist is that a surgeon can focus on surgical landmarks and manipulate the images for the purpose of surgical planning and decision making, whereas the radiologist focuses on the overall image presentation. The surgeon can virtually cut and manipulate the structure in any desired surgical angle countless times to achieve optimal perspectives for visualization of the pathology and pertinent anatomy (Figure 2).

**Figure 2:**
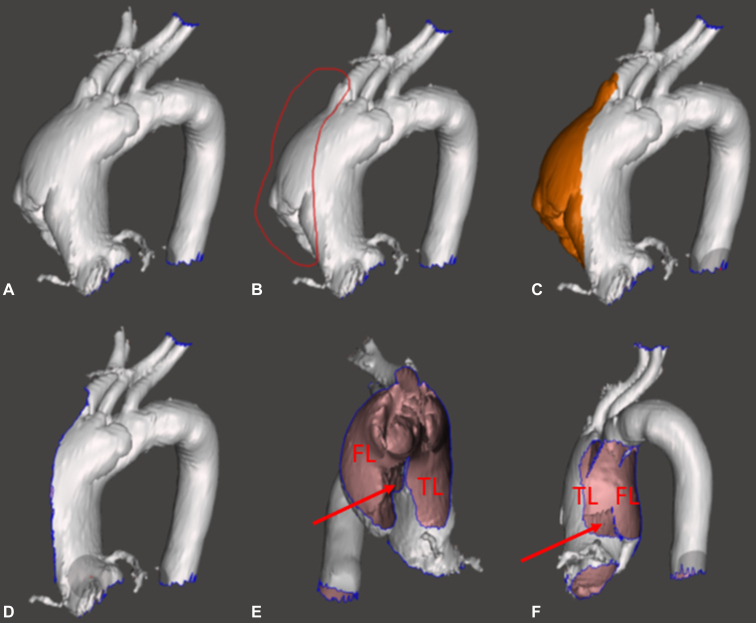
The process of virtual intravascular endoscopy to visualize the aorta from inside and outside and to manipulate it with desired cut and section planes. A, Aorta with deletion of all other structures. B, Free selection of an arbitrary cut plane with surgical importance to view the position of the dissection flap; the red line indicates the free-hand selection by the user. C, Once the area is selected and complete, the program automatically highlights it. D, By clicking DELETE, the marked area is removed. E, The geometry is freely rotated to view the inside from any angle of choice. F, The end result of a similar process to view from the *left side* of the aorta, illustrating the dissection flap (*arrow*) and its position, the true lumen (*TL*) and the false lumen (*FL*) (steps are not shown).

## Technique

The workflow is summarized in Figure 1 and the process is demonstrated in Video 1 and Table E1. The CT images (as DICOM files) are imported to InVesalius 3.1.1 (Renato Archer Information Technology Center) or any other medical image software such as Terrarecon (Terrarecon) or 3mensio (Pie Medical Imaging) (Table E2) that are available in hospitals performing structural heart procedures, and segmented to reconstruct patient-specific 3D meshes of the region of interest. The resulting 3D meshes are saved as .stl files. These files are similar to the 3D reconstructed images commonly provided with medical images and can be alternatively provided by radiology departments. The .stl files are then imported into Meshmixer 3.5 (Autodesk Inc) for 3D visualization and manipulation.

The open geometry in the Meshmixer can be cleaned from unnecessary structures with a simple point-and-click process. Optionally, the analysis tool of the program can automatically fix all geometry issues. Now, the geometry is ready for VIE. Surfaces of interest can be removed to review inside the aorta or heart chambers in any desired perspective. Not only can a surgeon manipulate the 3D structure in any direction, but a surgeon can also “travel” inside the aorta to obtain a realistic view of the anatomy and pathology.

## Comment

The utility of VIE in diagnosis and evaluation of TAAD has been previously studied.^1^^,^^2^ The supplemental benefit of VIE in providing detailed information on flaps and tears in TAAD and its complimentary role to routine CT scans have been reviewed in a large series by Qi and colleagues.^3^ Despite the clear benefits of VIE in TAAD, its widespread use is limited by 2 main factors: urgency of the cases and availability of the technical team. Exponential advances in computation power and availability of intuitive software have created an opportunity to harness the medical imaging technology with limited knowledge in the field.

We have demonstrated with ease how VIE is performed and how it assists in surgical planning for TAAD (Video 1). It allows a surgeon to autonomously visualize the plane of surgical interest, importantly nonlinear cut planes, even inside a vessel, rather than relying on a radiologist’s perspective, which uses the standard orthogonal planes only. This process is easily accomplished by Meshmixer, which effortlessly manipulates the anatomic meshes and has been used for cardiovascular simulation.^4^

This do-it-yourself process is efficient and can be used in emergency situations because it requires about 15 minutes from image upload to visualization. The programs outlined in this article are free-and-available programs that can be used on personal computers and the instructions are made for clinicians with basic computer knowledge.

## Conflict of Interest Statement

The authors reported no conflict of interest.

The *Journal* policy requires editors and reviewers to disclose conflicts of interest and to decline handling manuscripts for which they may have a conflict of interest. The editors and reviewers of this article have no conflicts of interest.

## Figures and Tables

**Table E1:** Step-by-step guide for virtual intravascular visualization

Step	Program used	Process
0 (First time only)		Installation of required programs (1-time installation only): 1. InVesalius: Install from https://invesalius.github.io/download.html 2. Meshmixer: Install from https://meshmixer.com/download.html
1	InVesalius For segmentation (For getting the 3D geometry from the CT scan images)	1. Open InVesalius program 2. Import medical images from the CT scan disc 3. Choose 1 of the image series with contrast and click import 4. On Mask Properties define the threshold 300-3500 and click create surface 5. The 3D geometry will appear in the window 6. Click export data and choose export 3S surface and save this 3D geometry file
2	Meshmixer (Autodesk) (For analysis and visualization)	1. Open Meshmixer program 2. Click on Import and choose the file that was saved. The 3D image will open and is ready. It can be rotated using the right mouse button 3. Click on select and with left mouse button select the unnecessary parts (eg, sternum or spine). By drawing the mouse, the selected areas will turn orange. Click the delete button on keyboard, they will disappear 4. Optional: Click on analysis and then inspector and then click on Autorepair All to automatically fix the errors in the images. This will improve the quality 5. Ready for visualization. Same as in step 3 any plane can be selected to remove to visualize inside the vessel of choice, and as in step 2, use the right button of the mouse to rotate the geometry freely in the space. Use the mouse wheel to zoom-in or to zoom-out. 6. Optional: The delete process can be reversed using the Undo button from Actions Tab, to make new manipulations

*3D*, 3 Dimensional; *CT*, computed tomography.

**Table E2:** A short list of software available for medical image analysis/segmentation

Software	Platform	Developer	Website
InVesalius	Free	Renato Archer Information Technology Center	https://invesalius.github.io/
3 mensio	Commercial	Pie Medical Imaging	https://www.3mensio.com
Terrarecon	Commercial	Terrarecon	https://www.terarecon.com
3D Slicer	Free	MIT and Brigham and Women’s Hospital	https://www.slicer.org/
TAVI Analysis	Commercial	GE Healthcare	https://www.gehealthcare.com
ITK SNAP	Free	US National Library of Medicine	http://www.itksnap.org
Mimics	Commercial	Materialise	https://www.materialise.com
MeVisLab	Free	MeVis Medical solutions	https://www.mevislab.de
Analyze	Commercial	Mayo Foundation	https://analyzedirect.com

## Supplementary Data

Video 1 The step- by- step process of virtual visualization of aorta. Video available at: https://www.jtcvs.org/article/S2666-2507(24)00146-9/fulltext.

## References

[1] Hornero F., Cervera V., Estornell J. (2005). Virtual vascular endoscopy for acute aortic dissection. Ann Thorac Surg.

[2] Sun Z., Cao Y. (2010). Multislice CT virtual intravascular endoscopy of aortic dissection: a pictorial essay. World J Radiol.

[3] Qi Y., Ma X., Li G., Ma X., Wang Q., Yu D. (2016). Three-dimensional visualization and imaging of the entry tear and intimal flap of aortic dissection using CT virtual intravascular endoscopy. PLoS One.

[4] Gerrah R., Haller S.J. (2022). Utilizing the fourth dimension for patient education in cardiovascular surgery. Ann Thorac Surg.

