# Supplementary figures and images for: Virtual intravascular visualization of the aorta for surgical planning in acute type A aortic dissection
Source: JTCVS Tech. 2024 Mar 28;25:28–32. doi: 10.1016/j.xjtc.2024.03.017 (PMC11184672; doi:10.1016/j.xjtc.2024.03.017)

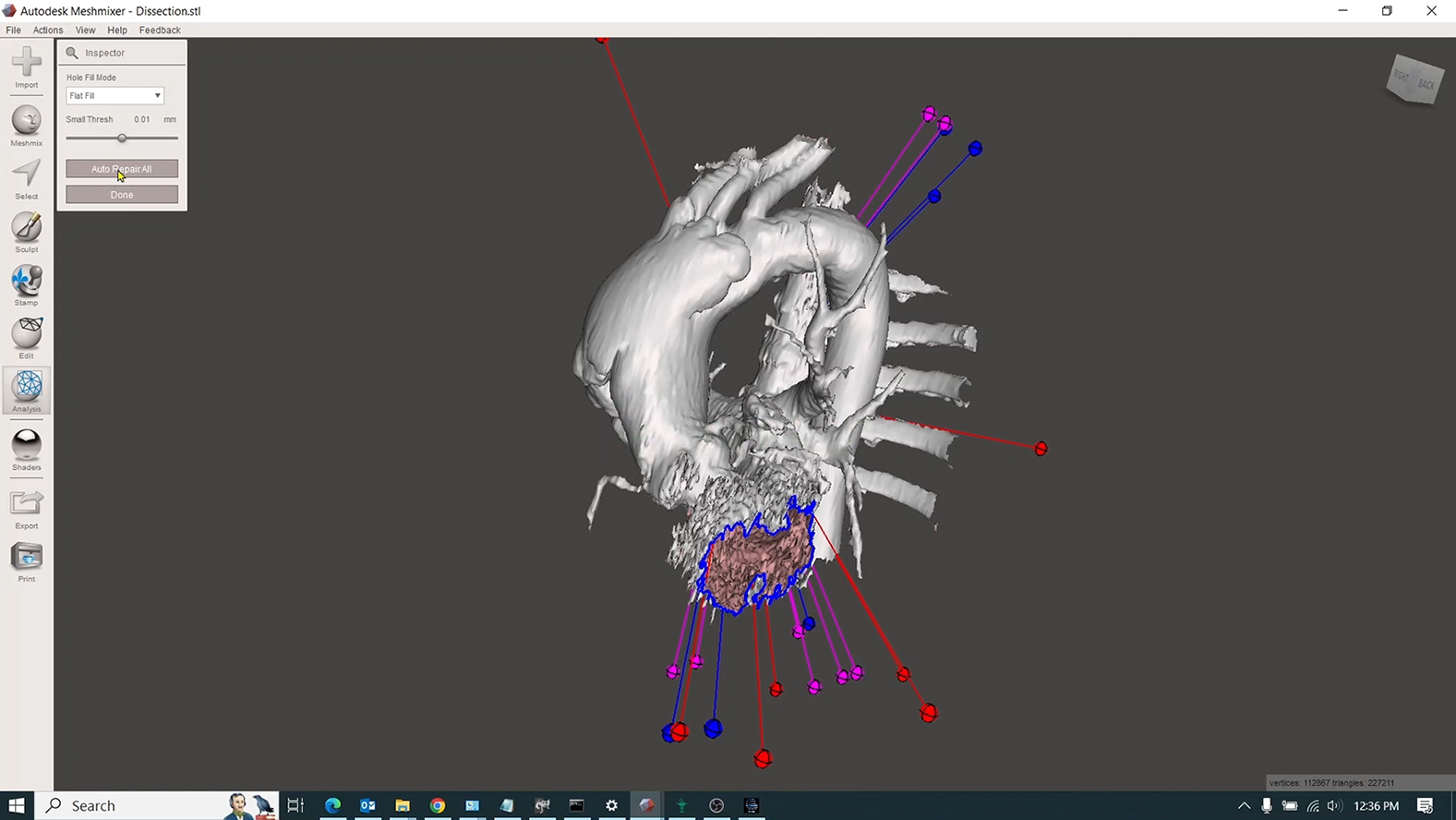

Supplement: Video 1 — The step- by- step process of virtual visualization of aorta. Video available at: https://www.jtcvs.org/article/S2666-2507(24)00146-9/fulltext. [file fx2.jpg]
